# Separating NADH and NADPH fluorescence in live cells and tissues using FLIM

**DOI:** 10.1038/ncomms4936

**Published:** 2014-05-29

**Authors:** Thomas S. Blacker, Zoe F. Mann, Jonathan E. Gale, Mathias Ziegler, Angus J. Bain, Gyorgy Szabadkai, Michael R. Duchen

**Affiliations:** 1 Centre for Mathematics and Physics in the Life Sciences and Experimental Biology, University College London, London WC1E 6BT, UK; 2 Research Department of Cell & Developmental Biology, University College London, London WC1E 6BT, UK; 3 Department of Physics and Astronomy, University College London, London WC1E 6BT, UK; 4 UCL Ear Institute, University College London, London WC1X 8EE, UK; 5 Department of Molecular Biology, University of Bergen, N-5008 Bergen, Norway; 6 Department of Biomedical Sciences, University of Padua and CNR Neuroscience Institute, Padua 35121, Italy; 7 These authors contributed equally to this work

## Abstract

NAD is a key determinant of cellular energy metabolism. In contrast, its phosphorylated form, NADP, plays a central role in biosynthetic pathways and antioxidant defence. The reduced forms of both pyridine nucleotides are fluorescent in living cells but they cannot be distinguished, as they are spectrally identical. Here, using genetic and pharmacological approaches to perturb NAD(P)H metabolism, we find that fluorescence lifetime imaging (FLIM) differentiates quantitatively between the two cofactors. Systematic manipulations to change the balance between oxidative and glycolytic metabolism suggest that these states do not directly impact NAD(P)H fluorescence decay rates. The lifetime changes observed in cancers thus likely reflect shifts in the NADPH/NADH balance. Using a mathematical model, we use these experimental data to quantify the relative levels of NADH and NADPH in different cell types of a complex tissue, the mammalian cochlea. This reveals NADPH-enriched populations of cells, raising questions about their distinct metabolic roles.

## Abstract (web summary)

NAD and NADP play fundamentally different roles in cellular metabolism, and yet these pyridine nucleotides cannot be distinguished spectroscopically in living cells. Blacker *et al.* demonstrate that fluorescence lifetime imaging can be used to quantify NADPH/NADH balance in cultured cells and in the mammalian cochlea.

## Graphical abstract

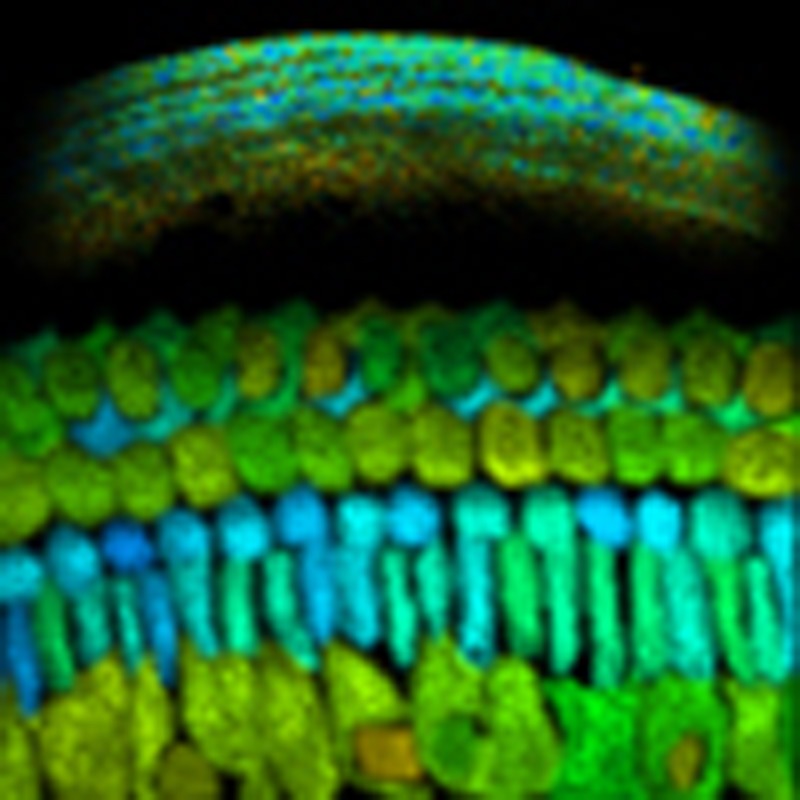

The cellular redox state is the central regulator of energy production and intermediary metabolism, playing a crucial role in health and disease^1^. The nicotinamide adenine dinucleotide (NAD^+^/NADH) and nicotinamide adenine dinucleotide phosphate (NADP^+^/NADPH) redox couples are the major determinants of redox state in the cell. However, these engage in distinct metabolic pathways. NAD drives ATP production in the cytosol by glycolysis and in the mitochondria by oxidative phosphorylation, while the phosphorylated analogue NADP governs lipid, amino acid and nucleotide biosynthetic pathways and the defence against reactive oxygen species by glutathione (GSH)^2^. Free radical generation is therefore determined by the redox state of NAD, while NADP redox state is key to antioxidant defence^3^. The relative abundance of the two pyridine nucleotides and their redox balance thus mediates cell fate in a wide range of diseases, including cancer, diabetes and neurodegeneration. Quantifying their behaviour is therefore essential in understanding the role of metabolism in these diseases. However, separating the contributions of the two pools in intact tissues has proven technically challenging^4^.

In the 1960s, Britton Chance *et al.*^5^ showed that live tissues illuminated with ultraviolet light emit blue fluorescence, arising primarily from mitochondrial NADH. The nicotinamide moiety of NADH absorbs light of wavelength 340±30 nm and emits fluorescence at 460±50 nm. As NADP is phosphorylated at a remote site of the molecule, the fluorescence properties of the nicotinamide ring of NADPH are identical to those of NADH^6^,^7^. Thus, changes in autofluorescence intensity may reflect changes in either [NADH] or [NADPH], often denoted as NAD(P)H to indicate the uncertain origin of the signal^8^,^9^.

Fluorescence lifetime imaging (FLIM) allows the study of NAD(P)H photochemistry inside living tissue^10^,^11^,^12^. This technique measures the rates of fluorescence decay of NAD(P)H, an excited-state process occurring over nanosecond time scales, which is highly sensitive to the immediate environment of the fluorophore. We have therefore investigated whether the fluorescence lifetime of cellular NADPH differs from that of cellular NADH, reflecting the different set of enzymes to which the two cofactors bind. Indeed, a substantial literature documents variations in the fluorescence lifetime of NAD(P)H in a range of physiological and pathological conditions^13^,^14^,^15^,^16^,^17^,^18^. However, despite the potential clinical applications of autofluorescence lifetime measurements, such as delineating the boundaries of accessible cancers^14^, the biochemical basis for these variations remains unknown. Moreover, the issue of whether FLIM may permit separation of NADH and NADPH fluorescence to resolve cellular specializations and dynamics in intact tissues is yet to be addressed. In the present study, we have explored the fluorescence decay properties of NADH and NADPH in living cells and tissues. Combined with computational and mathematical modelling, we have found that NAD(P)H fluorescence lifetime characteristics discriminate between NADH and NADPH. This provides a unique approach to identify cells within complex tissues that are enriched in NADPH, thus raising questions about their metabolic roles and specialization. We have also analysed the impact of altered metabolic state on NAD(P)H fluorescence decay characteristics, helping to place changes in lifetimes observed in transformed neoplastic cells on a firm biochemical footing.

## Results

### NAD(P)H fluorescence decay reflects bound NADPH/NADH ratio

We have previously shown that the fluorescence lifetime of NADPH is identical to that of NADH in solution^19^, demonstrating that fluorescence from the free, unbound pyridine nucleotides cannot be discriminated on the basis of their fluorescence lifetime. However, it has long been known that this value increases from 0.3–0.8 to 1–6.5 ns on binding to an enzyme, depending on the target to which the cofactor binds^10^,^20^, as well as by the simultaneous presence of substrate molecules^21^. Canonical NAD(P)H FLIM studies in live cells and tissues resolve two fluorescence lifetimes at each pixel; one of the order of 0.4 ns and the other larger and more variable at ~2 ns or more^11^,^12^,^22^. These represent the freely diffusing (*τ*_free_) and enzyme-bound NAD(P)H (*τ*_bound_) pools, respectively, confirmed by time-resolved anisotropy imaging studies^23^. As the distribution of bound NAD(P)H species will inevitably be more heterogeneous than implied by the single long lifetime, we suggest that *τ*_bound_ is a weighted mean of the fluorescence lifetimes of enzyme-bound species, a conclusion supported by computational modelling (see Supplementary Fig. 1a–m and Supplementary Note 1). It is primarily this parameter that has previously been observed to vary with changes in metabolism^11^,^24^,^25^,^26^.

**Figure 1:**
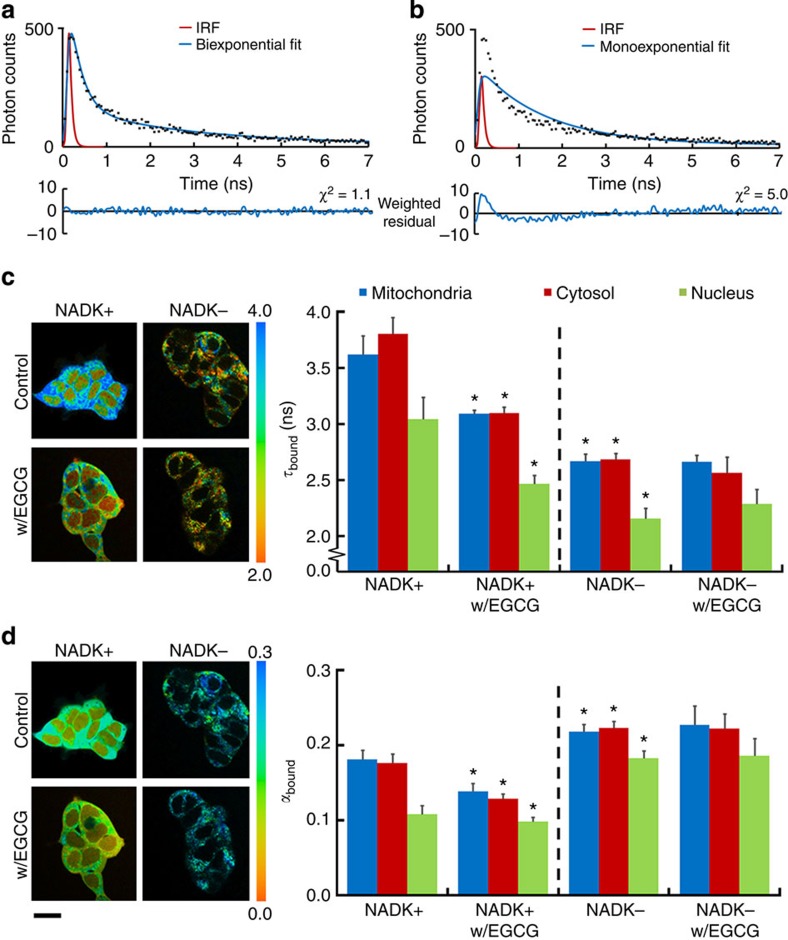
*τ*_bound_ reflects the enzyme-bound NADPH/NADH ratio in intact cells. (**a**,**b**) A biexponential decay model adequately described the NAD(P)H fluorescence decay measured in wtHEK293 cells (IRF, instrument response function). The mean 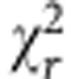 was 1.24±0.08 compared with 4±1 with a monoexponential fit (representative data from *n*=17 experiments). (**c**,**d**) Representative colour-coded images and mean *τ*_bound_ and *α*_bound_ values in NADK+ and NADK− HEK293 cells prior and following treatment with EGCG (100 μM), a competitive inhibitor of NADPH binding. Scale bar, 20 μm. Error bars indicate±s.d., *P<0.05 (two-tailed Student’s *t*-test, *n*=9).

Next, to understand the effect of varying [NADPH]/[NADH] ratios on *τ*_bound_, we acquired FLIM images of NAD(P)H in live HEK293 cells in which NADPH levels were genetically and pharmacologically manipulated. In wild-type cells, the fluorescence decays obtained at each pixel required a two-component model to yield good fits (Fig. 1a,b), as expected, with 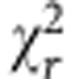 values close to unity. As such, addition of further components was not appropriate, to avoid inaccurate weightings due to overfitting^27^. Identical fluorescence decay parameters were observed in the cytosol and mitochondria, with mean (±s.d.) values of *τ*_bound_=2.7±0.2 ns, *α*_bound_=0.19±0.01 (fraction of bound NAD(P)H) and *τ*_free_=0.36±0.04 ns (Supplementary Table 1). Interestingly, *τ*_bound_ was significantly smaller in the nucleus at 2.3±0.2 ns (*P*=1E−8, two-tailed Student’s *t*-test, *n*=17 images).

To explain reported variations in *τ*_bound_, we hypothesized that, since NADH and NADPH are associated with different binding site structures inside the cell^28^, the measured *τ*_bound_ value could reflect the proportion of the two cofactors present. We therefore measured NAD(P)H fluorescence lifetimes in HEK293 cell lines in which NAD^+^ kinase (NADK) was either overexpressed (NADK+) or knocked down (NADK−)^29^. NADK is the key determinant of NADPH concentration inside the cell, and its overexpression results in a 10- to 15-fold higher [NADPH] in NADK+ relative to NADK− cells, leaving [NADH] relatively unaffected^29^. This was reflected by an NAD(P)H fluorescence intensity ~10-fold brighter in NADK+ compared with NADK− cells (Supplementary Fig. 2b). FLIM revealed that *τ*_bound_ was significantly larger in the NADK+ cells (*τ*_bound_=3.6±0.2 ns in the mitochondria, 3.8±0.2 ns in the cytosol) compared with the NADK− cells (*τ*_bound_=2.7±0.1 ns in both subcellular regions, *P*=1E−11 and *P*=5E−12, two-tailed Student’s *t*-test, *n*=9, Fig. 1c and Supplementary Table 1).

These data suggest that increased concentrations of bound NADPH result in increased values of *τ*_bound_. To verify this interpretation, we explored the impact of epigallocatechin gallate (EGCG) on the NAD(P)H FLIM parameters. EGCG is a potent competitive inhibitor of NADPH binding^30^ with no effect on NADH binding indicated by the BRENDA database^31^. Preferential competition for NADPH-binding sites by EGCG would decrease the bound NADPH population, leaving the bound NADH population unaffected. The value of *α*_bound_ should therefore be more sensitive to EGCG treatment in NADK+ cells than in NADK− cells, as bound NADPH will form a greater fraction of the total population of enzyme-bound NAD(P)H species. As predicted, *α*_bound_ decreased significantly from 0.18±0.02 to 0.13±0.02 in both the mitochondria and cytosol (*P*=4E−10, two-tailed Student’s *t*-test, *n*=9) on EGCG treatment in NADK+ cells but remained constant in NADK− cells, confirming the NADPH specificity of this compound (see Fig. 1d). Exposure to EGCG decreased *τ*_bound_ to 3.1±0.2 ns in both the mitochondria and cytosol of NADK+ cells (*P*=2E−12, two-tailed Student’s *t*-test, *n*=9) and to 2.5±0.2 ns in their nuclei (*P*=3E−7, two-tailed Student’s *t*-test, *n*=9), and did not significantly affect NADK− cells (Fig. 1c). In addition, treatment with this compound did not affect *τ*_free_ (Supplementary Fig. 2c,d). Thus, these data are consistent with a model where *τ*_bound_ reports the amount of enzyme-bound NADPH relative to bound NADH.

These data suggest that *τ*_bound_ can be used to quantify enzyme-bound NADPH/NADH ratios. We therefore developed a numerical model to quantify this phenomenon and generate predictions (see Supplementary Fig. 1k–o and Supplementary Note 2). By combining the enzyme-bound NAD(P)H fluorescence lifetimes measured in the NADK+ and NADK− cells, the biochemically quantified [NADH] and [NADPH] values in each cell line^29^ and a mathematical model in which NADH and NADPH were assumed to possess discrete and distinct fluorescence lifetimes when bound inside the cell, we found that *τ*_bound_ would describe the [NADPH]/[NADH] ratio by




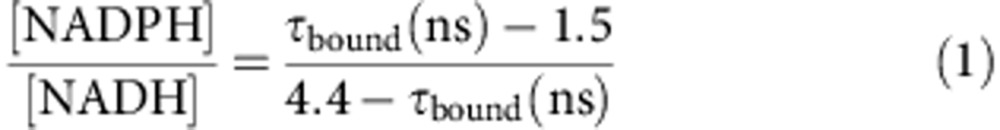




Application of this model to the NADK+ and NADK− data (see Supplementary Note 3) showed that the concentration of bound NADH remained constant on EGCG application, while the concentration of bound NADPH decreased ~3-fold with small differences in each subcellular compartment, supporting our hypothesis.

### Lifetime changes reflect mechanism of metabolic perturbation

In previous work^11^, shortening of *τ*_bound_ observed in tumours has been attributed to a shift from oxidative to glycolytic metabolism which occurs in many cancers, the so-called Warburg effect. To investigate this hypothesis in the light of the results described here, FLIM images of wild-type HEK293 cells were acquired following a range of manipulations that alter the balance of ATP production by aerobic or anaerobic pathways. Dependence on glycolysis was achieved by inhibition of mitochondrial oxidative phosphorylation using rotenone (10 μM) or uncoupling using FCCP (1 μM). The cells were driven to a more oxidative phenotype by inhibition of glycolysis by glucose deprivation in the presence of deoxyglucose (10 mM), while pyruvate (1 mM) or lactate (10 mM) were provided as mitochondrial-specific substrates.

**Figure 2:**
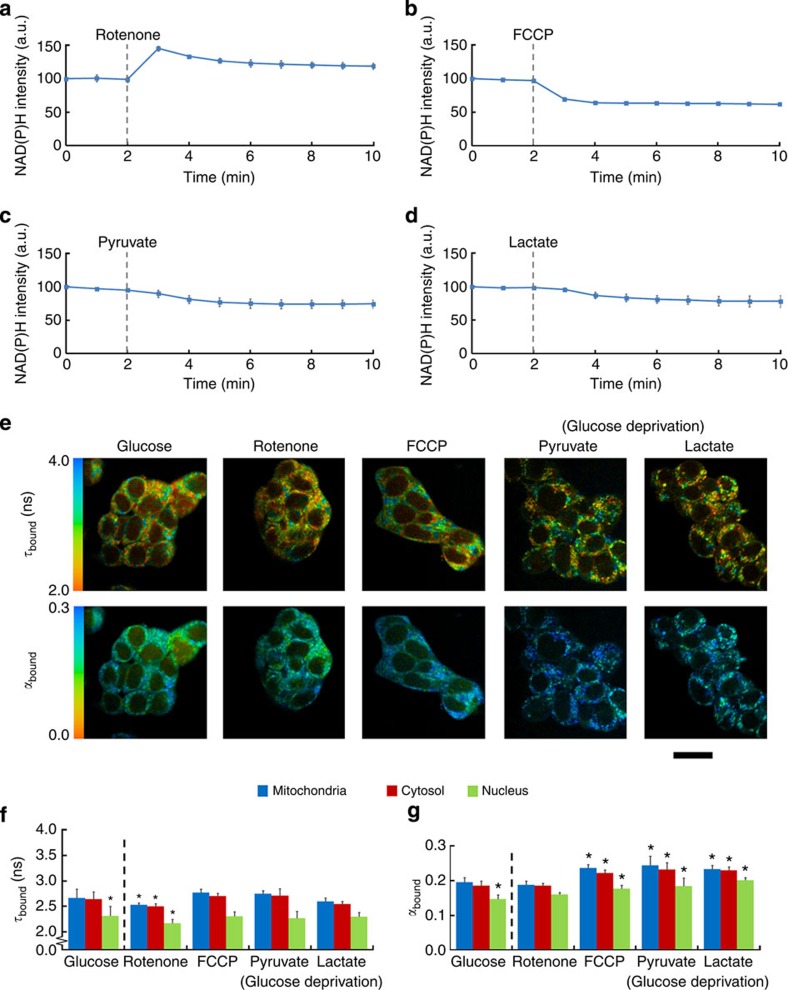
NAD(P)H fluorescence decay responses to metabolic perturbation are mechanism dependent. (**a**–**d**) Time series of NAD(P)H fluorescence intensity following treatments chosen to perturb oxidative (**a**: respiratory chain inhibition by rotenone, **b**: uncoupling with FCCP) and glycolytic metabolism (glucose replaced by deoxyglucose with **c**: pyruvate or **d**: lactate supplied as substrate). (**e**) Colour-coded images and (**f**,**g**) quantification of changes in *τ*_bound_ and *α*_bound_ on application of treatment. Scale bar, 20 μm. Error bars indicate±s.d., *P<0.05 (two-tailed Student’s *t*-test, *n*=9).

Ultraviolet confocal microscopy was used to establish the time taken for the redox state of the NAD(P) pool to reach a new steady state following each treatment (Fig. 2a–d). Inhibition of glycolysis decreased the cellular NAD(P)H fluorescence intensity by 26±6 and 22±8% with pyruvate and lactate supplied, respectively. Rotenone increased steady-state NAD(P)H fluorescence intensity by 20±5% while FCCP caused a decrease by 38±2% (each *n*=3). FLIM images were acquired at the steady-state fluorescence intensity levels following each treatment (Fig. 2e–g and Supplementary Table 2). Rotenone caused *τ*_bound_ to decrease significantly from 2.7±0.2 to 2.52±0.05 ns in both the mitochondria and cytosol (*P*=6E−4, two-tailed Student’s *t*-test, *n*=9), suggesting that this treatment caused the concentration of NADH present in the cell to increase relative to the concentration of NADPH, following inhibition of NADH oxidation by complex I. In contrast, *τ*_bound_ did not change in response to FCCP (*P*>0.05, two-tailed Student’s *t*-test, *n*=13). This lack of change in *τ*_bound_ suggested that uncoupling caused the oxidation of both NAD and NADP pools in equal measure. Increased oxidation of the NAD pool was to be expected on uncoupling due to the increased complex I activity, and the equal oxidation of the NADP pool was likely caused by the action of the mitochondrial transhydrogenase. In respiring mitochondria, this inner mitochondrial membrane protein transfers hydride from NADH to NADP^+^ powered by translocation of protons from the intermembrane space to the mitochondrial matrix^32^. However, on uncoupling, NADPH is oxidized and the hydride is passed to NAD^+^ to produce NADH^33^. Relative to controls, glucose deprivation in the presence of either pyruvate or lactate supply failed to significantly alter *τ*_bound_ (*P*>0.05, two-tailed Student’s *t*-test, both *n*=9). However, the mean value of *τ*_bound_ in both the mitochondria and cytosol of 2.70±0.01 ns in the presence of pyruvate was significantly larger than its value of 2.57±0.04 ns in the presence of lactate (*P*=7E−6, two-tailed Student’s *t*-test, *n*=9). As lactate dehydrogenase promotes NADH production during the conversion of lactate to pyruvate, the significantly smaller *τ*_bound_ value in the presence of lactate relative to pyruvate supports the interpretation developed in this work that this parameter reflects NADPH/NADH ratio, assuming NADPH production was identical in the presence of the two substrates.

While changes in *τ*_bound_, and thus the NADPH/NADH ratio, reflected the specific treatment causing a defect in OXPHOS or glycolysis, we observed that each treatment causing a net oxidation of the combined NAD(P) pools caused *α*_bound_ to increase (Fig. 2g). This is in support of previous suggestions that this parameter, reflecting the enzyme-bound population fraction of NAD(P)H, reports acute changes in the redox state of the cell^25^. However, confirming whether this parameter reflects phenotypic differences in the redox state of different cell types requires further investigation. Interestingly, each oxidizing treatment also caused an increase in *τ*_free_ (Supplementary Fig. 2e,f). However, as these lifetimes lie close to the time resolution of the FLIM system, such small differences may be an artefact of the fitting process, such as an interdependency between *τ*_free_ and *α*_bound_ (Supplementary Fig. 1j).

The lack of significant change in *τ*_bound_ in response to FCCP treatment or replacement of glucose with pyruvate or lactate, along with the very small, if significant, change in this parameter following rotenone treatment relative to the pathophysiological variations reported in the literature, suggests that changes in NAD(P)H fluorescence decay cannot be simply attributed to alterations in the balance between oxidative and glycolytic metabolism. Accordingly, no correlation between *τ*_bound_ and the balance of ATP production by glycolytic or oxidative means was observed by measuring the rates of oxygen consumption and lactate release in the wild-type, NADK+ and NADK− HEK293 cell lines (see Supplementary Fig. 3 and Supplementary Tables 3 and 4). Altogether, these data strongly suggest that any variation in the NAD(P)H fluorescence decay parameter *τ*_bound_ will be specific to the mechanism of metabolic perturbation, such as mitochondrial dysfunction caused either by respiratory chain inhibition or uncoupling, or the utilization of different substrates following inhibition of glycolysis. Such specificity has the potential to aid the study of the variety of possible metabolic rearrangements that may occur in cancer and other pathologies.

### FLIM reveals metabolic variations in complex tissues

The abundance of NADPH and NADH in live cells can be measured in tissues by biochemical means such as high performance liquid chromatography^29^,^34^. However, a microscopy-based imaging approach permits measurement of NADH and NADPH in discrete compartments within a cell or in different cell types within a complex tissue. Indeed, we have observed consistently smaller values of *τ*_bound_ in the nucleus compared with the rest of the cell. Combining enzyme localization data^31^ with the analysis and model provided here, NAD(P)H FLIM indicates that nuclear glucose metabolism favours NADH-producing glycolysis rather than NADPH-producing pentose phosphate pathway (PPP), resulting in a more oxidized NADP pool in this region (see Supplementary Note 4).

**Figure 3:**
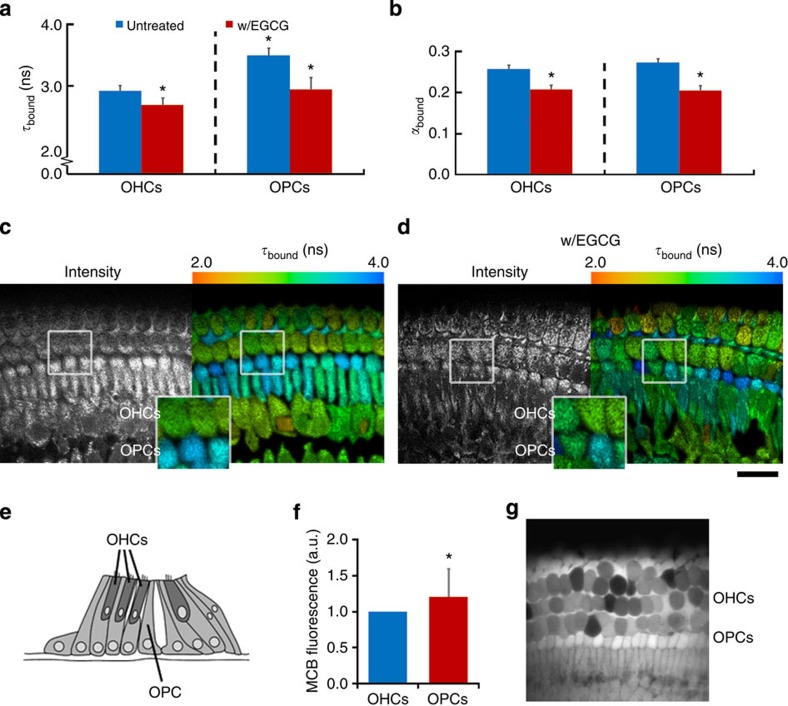
Supporting cells in the mammalian cochlea exhibit increased enzyme-bound NADPH. (**a**) Mean *τ*_bound_ and (**b**) *α*_bound_ values in outer hair cells (OHC’s) and adjacent outer pillar ‘supporting’ cells (OPC’s) under control conditions and following application of EGCG (200 μM). Error bars indicate±s.d., *P<0.05 (two-tailed Student’s *t*-test, *n*=11). (**c**,**d**) Corresponding representative FLIM images colour coded for the mean parameter value in each cell. Scale bar, 25 μm. (**e**) Schematic diagram showing organ of Corti in the cochlear explants, indicating the positions of OHC’s and OPC’s. (**f**) Mean fluorescence intensity in OPC’s and OHC’s. Error bars indicate±s.d., *P<0.05 (Wilcoxon signed-rank test, *n*=17). (**g**) Representative image following MCB staining for GSH concentration.

To explore the application of NAD(P)H FLIM in multicellular specimens, the redox metabolism of the mammalian cochlea was investigated. The cochlea is a structure containing a highly ordered system of functionally distinct cell types, which are easily identifiable from their architecture (Fig. 3e). FLIM imaging of cochlear explant cultures revealed that *τ*_bound_ differed markedly between different cell types. The glia-like outer pillar ‘supporting’ cells (OPCs) exhibited an extended fluorescence lifetime of 3.5±0.1 ns, while the mean value of *τ*_bound_ measured in the neighbouring sensory receptors, the outer hair cells (OHCs), was significantly lower at 2.9±0.1 ns (*P*=2E−15, two-tailed Student’s *t*-test, *n*=19, Fig. 3a–d). EGCG treatment decreased *τ*_bound_ in supporting cells by 0.6±0.2 ns (*P*=1E−6, two-tailed Student’s *t*-test, *n*=11) to a mean of 2.9±0.2 ns, but by only 0.2±0.2 ns (*P*=8E−4, two-tailed Student’s *t*-test, *n*=11) to a mean of 2.7±0.1 ns in hair cells (Supplementary Table 5). These data confirm that the differences in fluorescence lifetime between cell types were a measure of differences in the bound NADPH/NADH ratio.

Application of the mathematical model developed in this work (Supplementary equations 10 and 11) implied that the total concentration of reduced pyridine nucleotides were equal in the OHCs and OPCs. In the OHCs, the absolute concentrations of NADH and NADPH were equal at 1±0.2 a.u. However, in the OPCs, the concentration of NADPH was significantly larger at 1.4±0.3 a.u. (*P*=4E−4, Wilcoxon signed-rank test, *n*=19), with a correspondingly smaller concentration of NADH (0.7±0.2 a.u., *P*=2E−4, Wilcoxon signed-rank test, *n*=19). To assess the functional significance of this finding, we measured the distribution of GSH in the tissue using monochlorobimane (MCB) staining, since a major demand for NADPH in glial cells arises from GSH turnover^35^. The glia-like OPCs showed significantly higher [GSH] compared with OHCs (*P*=0.02, Wilcoxon signed-rank test, *n*=17, Fig. 3f,g), consistent with a functional requirement for NADPH enrichment. Thus, our interpretation of *τ*_bound_ is consistent with alternative indicators of cellular redox state.

## Discussion

This work shows that FLIM can be used effectively to differentiate between NADH and NADPH at the level of the single cell or organelle. The results suggest that enzyme-bound NADPH possesses a significantly larger fluorescence lifetime than enzyme-bound NADH within the cellular environment, so that the proportion of enzyme-bound NADPH and NADH present in live tissue determines the lifetime of their combined fluorescence decay. By making the simplifying assumption that bound NADH and bound NADPH possess finite and distinct fluorescence lifetimes inside the cell, the relative contribution of each cofactor to the combined fluorescence signal could be calculated. With excitation at 700 nm and a 435–485 nm detection window, the intracellular fluorescence lifetimes of NADH and NADPH were predicted to be 1.5±0.2 and 4.4±0.2 ns respectively. It is reasonable to hypothesize that these conclusions can be extended to all cell types as the fluorescence lifetime of NAD(P)H when bound to an enzyme is determined by its local environment in the binding site^21^, and the NADH and NADPH-binding sites are two of the most highly conserved in all biology^36^,^37^. Indeed, we have observed values of *τ*_bound_ that are similar in magnitude across a range of cell types, including isolated ventricular cardiomyocytes and neurons in culture or in brain slices (data not shown).

The analysis and model presented herein are consistent with previously published NAD(P)H FLIM studies. In 2008, Niesner *et al.*^38^ performed a novel study on the decay of NAD(P)H fluorescence in granulocytes in the presence of *Aspergillus fumigatus* fungus. The parameter *τ*_bound_ was ~2 ns within the bulk cytosol of the granulocytes. However, localized subplasmalemmal regions of the cytosol in contact with the fungus displayed increased values of 3.7 ns. This was attributed to a unique fluorescence lifetime of NADPH when bound to the NADPH oxidase activated in response to pathogenic exposure. However, the computational simulations performed here showed that *τ*_bound_ is a weighted average of the fluorescence lifetimes of the enzyme-bound NAD(P)H species present. For NADPH oxidase alone to cause an increase in *τ*_bound_ as large as that observed in the granulocytes would require this enzyme to be present at a greater concentration than the NADH-binding enzymes of the cytosol. The Model Organism Protein Expression Database (MOPED)^39^ within the GeneCards human gene compendium^40^ shows that expression of glyceraldehyde 3-phosphate dehydrogenase outweighs that of the NADPH-binding subunit of NADPH oxidase (neutrophil cytosolic factor 2)^41^ by around 100 to one in neutrophils. It is therefore more likely that the NAD(P)H fluorescence lifetime observed in the regions of the cytosol where NADPH oxidase was activated was due to increased local NADPH production, as implied from the results reported here. Indeed, the large quantities of superoxide produced by NADPH oxidase requires plentiful supply of NADPH. Activation of this enzyme is thus associated with increased flux of glucose through the PPP^42^.

Changes in the fluorescence decay of NAD(P)H have recently been observed in applications ranging from wound healing^17^ to stem cell differentiation^18^ and necrotic deterioration of skin^15^ to staurosporine-induced apoptosis^16^. The large number of studies reporting differences between the fluorescence lifetime of NAD(P)H in healthy control cells and cells at different stages of carcinogenesis^11^,^43^,^44^ have prompted the design of clinical instruments for the detection, diagnosis and staging of accessible tumours using time-resolved autofluorescence measurements^14^,^45^,^46^,^47^,^48^,^49^,^50^. While the Warburg effect is the most well known of the metabolic shifts occurring during cancer development, tumorigenesis is also associated with variations in glucose flux through the PPP and various biosynthetic pathways utilizing NADPH^51^. As none of the severe pharmacological perturbations to cytosolic or mitochondrial ATP production applied in this work could reproduce responses in *τ*_bound_ of the magnitude caused by an increased NADPH/NADH ratio, our results strongly suggest that NAD(P)H fluorescence lifetime differences observed between healthy and pathological states reflect shifts in the NADPH/NADH balance.

In this work, termination of oxidative phosphorylation by uncoupling and of glycolysis by glucose deprivation could not be resolved on the basis of the fluorescence decay parameters measured under these conditions. In addition, two of the cell lines studied here with similar fluorescence decay parameters were shown to differ in their reliance on aerobic and anaerobic metabolism (wild-type HEK293 and NADK−). This implies that changes in the NAD(P)H fluorescence decay do not simply report shifts between an oxidative or glycolytic phenotype, but reflect the differential response of the NAD and NADP pools following a metabolic alteration. For example, both uncoupling and electron transport chain inhibition terminate aerobic ATP production yet have opposing effects on the overall NAD(P) redox state and thus, perhaps on *α*_bound_. Changes in *τ*_bound_ will be induced by metabolic transitions that cause divergent effects on NAD- and NADP-associated pathways. Thus, neither parameter alone will permit the detection of oxidative or glycolytic states using NAD(P)H fluorescence decay measurements. However, a method for assessing the pathways used for ATP production by measuring autofluorescence may be possible by combining a number of observables, including *α*_bound_ and *τ*_bound_, alongside fluorescence intensity information and similar measurements from flavoprotein fluorescence as part of a spectrally resolved lifetime imaging tool^24^.

In the present study, we have investigated the canonical form of FLIM in which fluorescence lifetimes *τ*_free_ and *τ*_bound_, along with the weighting parameter *α*_bound_, are extracted by least-squares fitting of a biexponential decay at each pixel^11^,^24^,^25^,^26^. Drawbacks of this method of analysis have been identified, such as the computational burden of fitting 10^4^–10^5^ independent decay curves in a single image, difficulties in resolving decay components with closely spaced lifetimes and correlations between the fit parameters^52^. Other groups have therefore focussed on developing novel analytical techniques. For example, Yaseen *et al.*^53^ recently applied global analysis to the application of NAD(P)H FLIM of the rat cortex. In this approach, the lifetimes of four decay components were shared across the image and a novel computational algorithm recovered the optimum amplitudes of each species to describe the fluorescence decay at each pixel. Interestingly, two enzyme-bound components were identified in the tissue with lifetimes of 1.7 and 3.2 ns, perhaps corresponding to enzyme-bound NADH and NADPH. Another approach to which our conclusions may be strongly applicable is the phasor method developed by Digman *et al.*^52^ Here, the real and imaginary parts of the Fourier transform of the fluorescence decay at each pixel of a FLIM image define the coordinates of a point in a two-dimensional phase space. The relative abundance of two or more fluorescent species with different lifetimes can then be inferred from the location of the pixels in the phasor plot. Application of this ‘fit-free’ approach to separating NADH and NADPH fluorescence will be the subject of further work.

Altogether, we have shown that the fluorescence lifetime characteristics of NAD(P)H in live cells and tissues can be used to discriminate between NADH and NADPH fluorescence, providing, for the first time, a biochemical framework for interpretation of NAD(P)H FLIM studies. Such a technique permits the separation of NADH and NADPH redox signalling without disrupting the sample on the addition of external probes^54^,^55^, allowing complex tissue preparations to be investigated. The approach revealed previously unknown cellular metabolic specializations in the mammalian cochlea, highlighting a subpopulation of cells characterized by high NADPH levels, opening up new avenues of research to understand the functional significance of redox pathways with respect to the physiological roles of these cells.

## Methods

### Cell culture

HEK293 cells were obtained from the American Type Culture Collection and grown in advanced Dulbecco’s modified Eagle Medium (Gibco) supplemented with 10% fetal bovine serum, 2 mM GlutaMAX, 100 U ml^−1^ penicillin and 100 μg ml^−1^ streptomycin (Gibco). Production of the NADK+ and NADK− cell lines has been reported previously^29^. These cultures were additionally supplemented with 0.1 mg ml^−1^ G-418 selective antibiotic (Gibco). All cells were grown as monolayers in sterile 75 cm^2^ tissue culture flasks (Thermo Scientific Nunc) in a 37 °C, 5% CO_2_ incubator.

### Cochlea explant cultures

Cochlear coils were isolated from male and female post-natal day 2–3 Sprague Dawley rats as previously described^56^. Briefly, auditory bullae were removed and transferred into Medium 199 with Hank’s balanced salts (Life Technologies). The cartilaginous wall of the bulla was opened and the whole cochlea extracted. The stria vascularis and Reissner’s membrane were removed and the cochlea cut into three coils. The cochlear coils were placed onto Cell-Tak cell and tissue adhesive (BD Biosciences)-coated dishes (MatTek). For coating, cell adhesive was diluted to 70 μg ml^−1^ in 0.1 mM NaHCO_3_. The cochlear explants were incubated overnight in DMEM/F12 (Gibco), supplemented with 1% fetal bovine serum (Life Technologies) in a 37 °C, 5% CO_2_ incubator. The isolation was performed in accordance with the United Kingdom Animals (Scientific Procedures) Act of 1986 and in compliance with the Biological Services Management Group and the Biological Services Ethical Committee, University College London.

### Live-cell microscopy

On the microscope stage, coverslips containing 300,000 cells were maintained at 37 °C in a metal ring and bathed in DMEM solution at pH 7.4 containing 10 mM HEPES and 2 mM GlutaMAX. Glucose (25 mM) was present under control conditions and during EGCG treatment (100 μM in cell lines, 200 μM for cochlea). Pharmacological perturbations to metabolism were applied by the dropwise addition of working concentrations of each compound, diluted from stock solutions in DMEM recording medium.

NAD(P)H FLIM was performed on an upright LSM 510 microscope (Carl Zeiss) with a 1.0 NA × 40 water-dipping objective using a 650-nm short-pass dichroic and 460±25 nm emission filter. Two-photon excitation was provided by a Chameleon (Coherent) Ti:sapphire laser tuned to 700 nm, with on-sample powers kept below 10 mW. Spectral controls (see Supplementary Fig. 4 and Supplementary Note 5) confirmed the NAD(P)H specificity of this excitation wavelength and emission filtering. Photodamage controls (see Supplementary Tables 6 and 7 and Supplementary Note 6) demonstrated that FLIM parameters were not varying over the course of imaging. Emission events were registered by an external detector (HPM-100, Becker & Hickl) attached to a commercial time-correlated single photon counting electronics module (SPC-830, Becker & Hickl) contained on a PCI board in a desktop computer. Scanning was performed continuously for 4 min with a pixel dwell time of 1.6 μs. To identify mitochondrial and cytosolic regions, the mitochondrially targeted fluorescent dye tetramethylrhodamine methyl ester (TMRM) was added to the recording medium, at a final concentration of 25 nM, 20 min before imaging. TMRM fluorescence was collected for a 10-s burst using a 610±30 nm emission filter. Excitation was provided at the same wavelength as NAD(P)H to avoid possible chromatic aberration. The 585±15 nm emission spectrum of TMRM ensured its fluorescence did not contaminate the NAD(P)H FLIM images.

NAD(P)H fluorescence intensity time series and MCB imaging were performed on an inverted LSM 510 laser scanning confocal microscope (Carl Zeiss) with 351 nm illumination from an argon ion laser (Coherent Enterprise UV). MCB (30 min loading at 50 μM) and NAD(P)H fluorescence were observed using a 351-nm long-pass dichroic and 460±25 nm band-pass emission filter with a × 40, 1.3 NA quartz oil immersion objective. Images (12-bit 512 × 512) were obtained with a pixel dwell time of 1.6 μs. Time series measurements were obtained at 1 min intervals. To reduce noise, the image recorded at each time point was a mean of four consecutive scans. Fluorescence intensity levels were extracted using ImageJ (NIH).

### Metabolic controls

For the measurement of oxygen consumption, cells were trypsinized and resuspended at ~1 million cells per ml in DMEM buffered with 10 mM HEPES and supplemented with 25 mM glucose and 2 mM GlutaMAX. Respiration rates were measured in triplicate at 37 °C with the high-resolution Oxygraph (Oroboros Oxygraph-2k). State 4 respiration values were obtained in the presence of 2.5 μM oligomycin, maximal oxidative capacities were determined in the presence of 2 μM FCCP and non-mitochondrial background oxygen consumption was determined in the presence of 2.5 μM antimycin A. Lactate release rates were measured in triplicate by removing a sample of serum-free DMEM from 70% confluent cell cultures at 1 h intervals for 5 h total and determining the concentration of lactate present using a commercially available plate-reader assay (Sigma Aldrich) in absorption mode. For normalization of both oxygen consumption and lactate release rates, final cell counts were performed using a haemocytometer.

### FLIM data analysis

Following 5 × 5 binning of photon counts at each pixel, fluorescence decay curves of the form




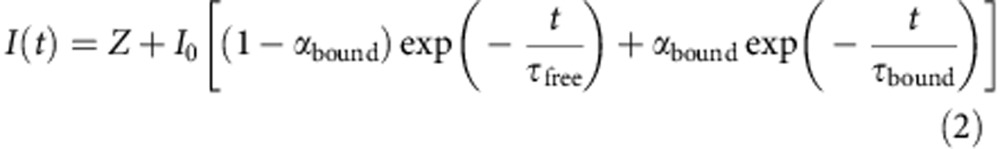




were fit to the FLIM images using iterative reconvolution in SPCImage (Becker & Hickl), where *Z* allows for time-uncorrelated background noise. The instrument response function of the FLIM system was obtained by measuring the fluorescence decay profile of second harmonic generation by a potassium dihydrogen phosphate (KDP) crystal at 920 nm, grown by leaving a molar solution of KDP in water on a coverslip to evaporate. Matrices of the fit parameters *τ*_free_, *α*_bound_ and *τ*_bound_, along with the total photons counted at each pixel, were exported from SPCImage. In MATLAB (The Mathworks), a 16-bit grayscale image was produced for each parameter matrix in which the intensity of each pixel was proportional to the parameter value at that location. ImageJ was then used to measure the grayscale intensity in the parameter images and the values obtained converted back to parameter values using the scaling factors applied in their production. Masks identifying the location of mitochondrial and cytosolic pixels were created by importing images of the TMRM distribution in the cell and a nuclear mask was defined by hand, allowing the parameters describing the fluorescence decay of NAD(P)H in each of these regions to be extracted separately (Supplementary Fig. 2a).

### Statistical analysis

NAD(P)H fluorescence lifetime parameters obtained using FLIM are reported as a mean over at least three regions of at least three separate cultures. Uncertainties in these values were taken as the s.d. of the measurements. Differences between the lifetime parameters measured under different conditions were tested for statistical significance (*P*<0.05) using a two-tailed Student’s *t*-test. Statistically significant differences between data sets normalized to be expressed in relative arbitrary units (fluorescence intensity, NADH/NADPH concentration) were assessed using a Wilcoxon signed-rank test. For the estimation of uncertainties in the predictions of the numerical model, the standard formula for the calculation of the error 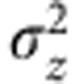 in an arbitrary function *Z*=*Z*(*A*, *B*, *C*,...) was applied,




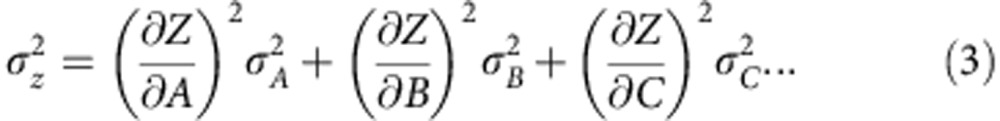




## Authors contributions

M.Z. provided the NADK+ and NADK– cell lines. J.E.G. prepared cochlea cultures and oversaw their imaging. Z.F.M., G.S. and M.R.D. performed preliminary experiments. T.S.B. performed the experiments, data analysis and modelling. A.J.B., G.S. and M.R.D. supervised the work. T.S.B., G.S. and M.R.D. wrote the manuscript.

## Additional information

**How to cite this article:** Blacker, T. S. *et al.* Separating NADH and NADPH fluorescence in live cells and tissues using FLIM. *Nat. Commun.* 5:3936 doi: 10.1038/ncomms4936 (2014).

## Supplementary Material

Supplementary Information Supplementary Figures 1-4, Supplementary Tables 1-7, Supplementary Notes 1-6 and Supplementary References
