# Supplementary material for: Separating NADH and NADPH fluorescence in live cells and tissues using FLIM
Source: Nat Commun. 2014 May 29;5:3936. doi: 10.1038/ncomms4936 (PMC4046109; doi:10.1038/ncomms4936)
Supplement: Supplementary Information — Supplementary Figures 1-4, Supplementary Tables 1-7, Supplementary Notes 1-6 and Supplementary References [file ncomms4936-s1.pdf]

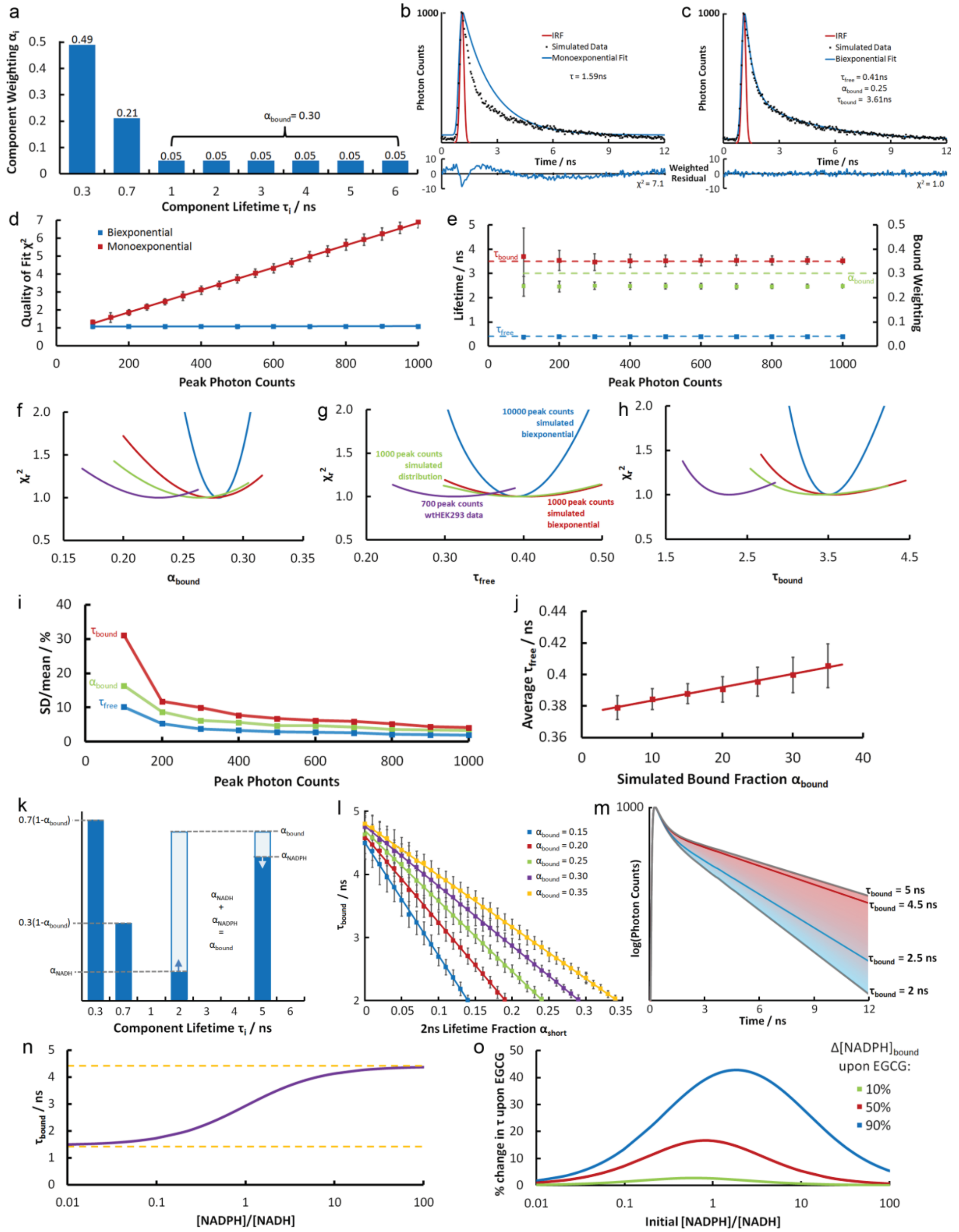

Supplementary Figure 1: Development of a numerical model for τ_{bound}. With an underlying distribution of 8 fluorescence lifetimes (a), the simulated NAD(P)H fluorescence decay was best fit to a two-component model across the range of signal levels obtainable using FLIM of live tissue (b-d). τ_{free} and τ_{bound} provided weighted means of the free and enzyme-bound populations respectively (e), although shallowing of the support plane of each parameter (f-h) caused the spread of extracted parameter values to increase at lower (experimental) signal levels (i). Additionally, a parameter correlation was observed between τ_{free} and α_{bound} (j). τ_{bound} varied linearly as the proportion of two bound species with different lifetimes were varied at constant α_{bound} (k-m), suggesting a nonlinear dependency of τ_{bound} on the bound NADPH/NADH ratio (n-o).

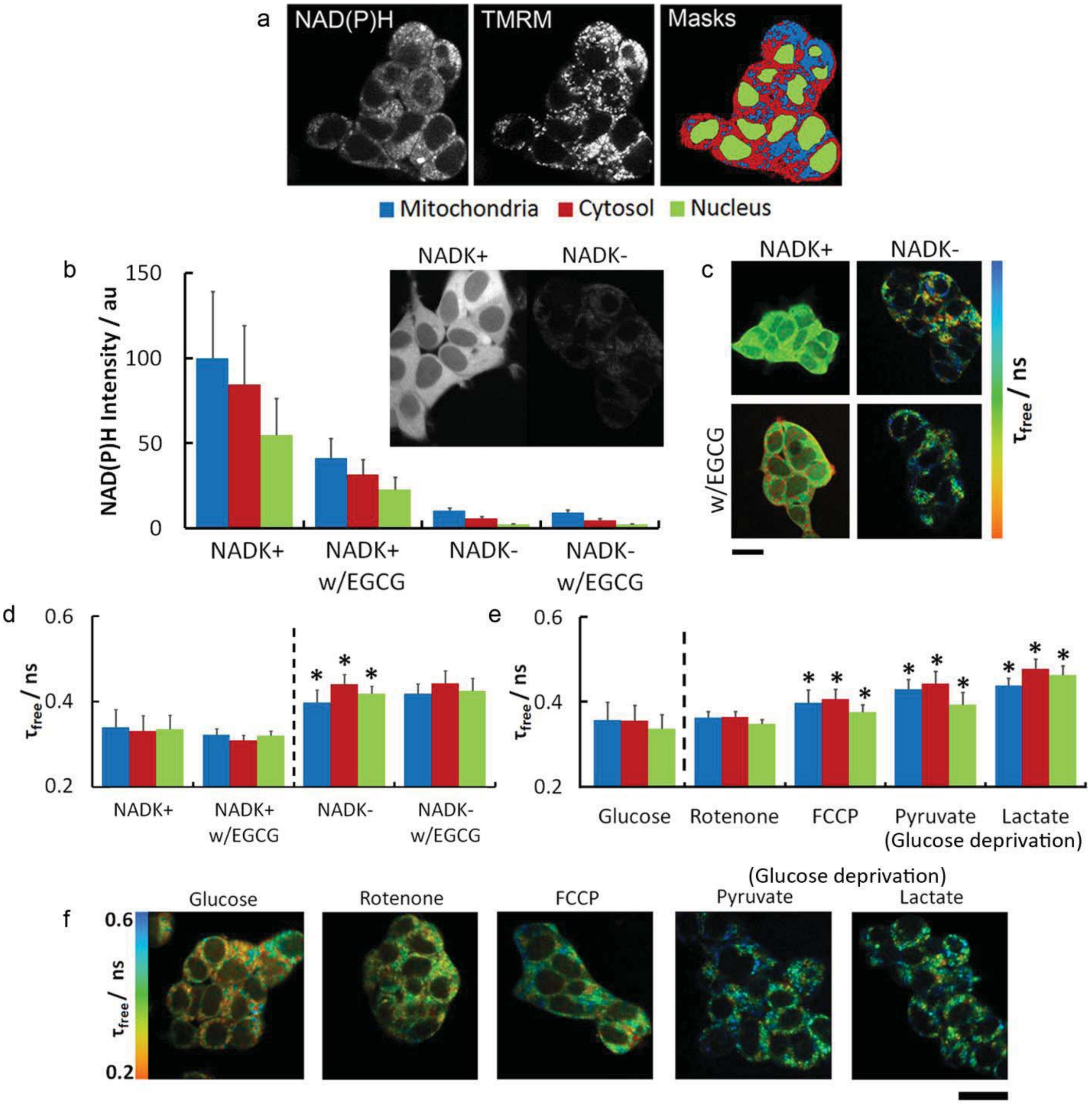

Supplementary Figure 2: Additional data extracted from NAD(P)H FLIM of live HEK293 cells. Fluorescence decay parameters were resolved into mitochondrial, cytosolic and nuclear compartments using masks defined by TMRM and NAD(P)H fluorescence intensity (a). The increased [NADPH] caused by NADK overexpression was clear from the bright NAD(P)H fluorescence in these cells (b). τ_{free} was larger in NADK- cells than in NADK+ cells (c-d). However, as these lifetimes lie close to the time resolution of the FLIM system, such small differences could be an artefact of the fitting process. Indeed, in wtHEK293's (e-f), τ_{free} remained constant upon respiratory chain inhibition with rotenone and increased following all treatments in which the NAD(P)H intensity decreased and α_{bound} increased (respiratory chain uncoupling using FCCP and glucose deprivation while supplying pyruvate or lactate as metabolic substrates), perhaps indicating a parameter correlation. Scale bars 20 μm.

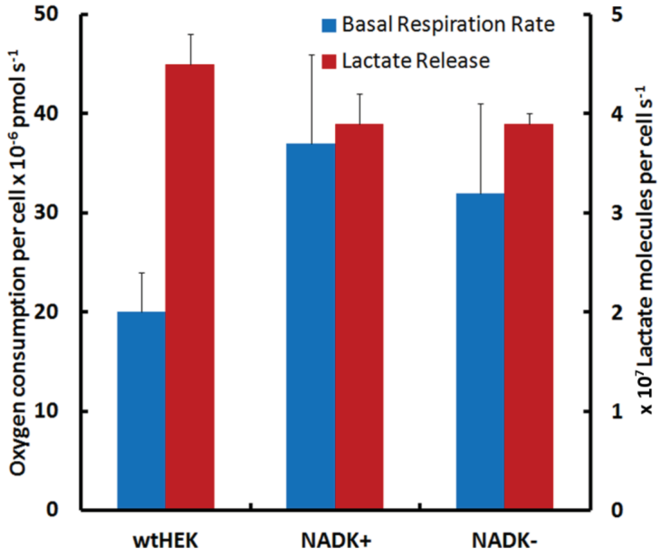

Supplementary Figure 3: Routine oxygen consumption and lactate release rates of wild-type HEK293 cells and HEK293 cells with NAD kinase overexpressed (NADK+) or knocked down (NADK-). Perturbing the expression of NAD kinase did not change the balance of ATP production between oxidative and glycolytic means, with equal oxygen consumption and lactate release rates in the NADK+ and NADK- cells. However, wild-type HEK293 cells, from a separate lineage from those used to produce the mutant cell lines, showed increased reliance on glycolysis (40±20% smaller oxygen consumption rate and a 15±9% larger lactate release rate than the NADK- cells). The NAD(P)H fluorescence decay parameters of the NADK+ and NADK- cells were dramatically different, while those of the wild-type HEK293 cells were similar to those of the NADK- cells. This implies that variations in the fluorescence decay of NAD(P)H do not simply reflect differences in the reliance on aerobic or anaerobic energy metabolism.

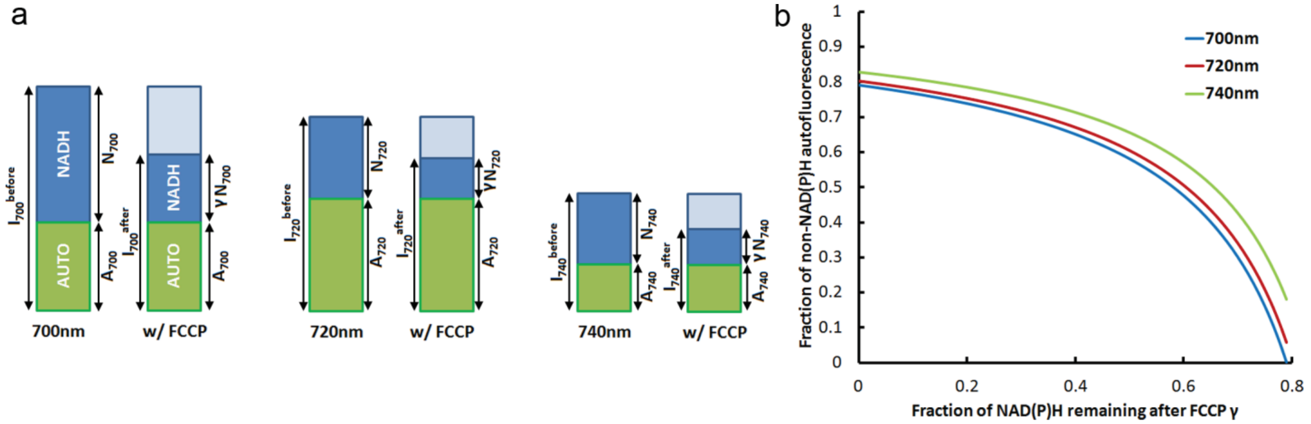

Supplementary Figure 4: Spectral control experiments. To confirm that excitation at 700nm, the peak of the two-photon cross section spectrum of NADH, did not cause increased autofluorescence compared to other potential excitation wavelengths, the scheme shown in (a) was devised. Application of FCCP caused the NAD(P)H-specific signal to drop to a fraction γ of its initial value. While the initial contribution to the total measured fluorescence intensity of NAD(P)H and other autofluorescence may have differed across the wavelengths, γ remains constant. This allowed the levels of autofluorescence at 700nm, 720nm and 740nm to be plotted as a function of all potential values of this unknown parameter (b). For all γ, the autofluorescence levels at 700nm were smaller than those at 720nm and 740nm, confirming the validity of this wavelength for efficient excitation of NAD(P)H.

	τ_{free} (ns)			α_{bound}			τ_{bound} (ns)		
	M	C	N	M	C	N	M	C	N
wtHEK	0.36	0.36	0.34	0.20	0.19	0.15	2.7	2.7	2.3
±	0.04	0.04	0.03	0.01	0.01	0.01	0.2	0.1	0.2
NADK+	0.34	0.33	0.34	0.18	0.18	0.11	3.6	3.8	3.0
±	0.03	0.03	0.03	0.01	0.01	0.01	0.2	0.2	0.1
w/EGCG	0.32	0.31	0.32	0.13	0.13	0.10	3.1	3.1	2.5
±	0.02	0.01	0.01	0.02	0.02	0.01	0.2	0.2	0.2
NADK-	0.40	0.44	0.42	0.22	0.22	0.18	2.67	2.7	2.2
±	0.03	0.04	0.04	0.02	0.02	0.02	0.06	0.1	0.2
w/EGCG	0.42	0.44	0.42	0.23	0.22	0.19	2.7	2.6	2.3
±	0.02	0.03	0.04	0.02	0.02	0.02	0.1	0.2	0.2

Supplementary Table 1: NAD(P)H fluorescence decay parameters in mitochondrial (M), cytosolic (C) and nuclear (N) compartments in wild-type and genetically modified HEK293 cell lines.

	τ_{free} (ns)			α_{bound}			τ_{bound} (ns)		
	M	C	N	M	C	N	M	C	N
Glucose	0.36	0.36	0.34	0.19	0.19	0.15	2.7	2.7	2.3
±	0.04	0.04	0.03	0.01	0.01	0.01	0.2	0.1	0.2
Rotenone	0.36	0.36	0.35	0.19	0.19	0.16	2.53	2.51	2.17
±	0.01	0.01	0.01	0.01	0.01	0.01	0.03	0.05	0.07
FCCP	0.40	0.41	0.38	0.24	0.22	0.18	2.77	2.70	2.31
±	0.03	0.02	0.02	0.01	0.01	0.01	0.07	0.05	0.09
Pyruvate	0.43	0.44	0.39	0.24	0.23	0.18	2.75	2.7	2.3
±	0.02	0.03	0.03	0.03	0.02	0.02	0.06	0.1	0.1
Lactate	0.44	0.48	0.46	0.23	0.23	0.20	2.60	2.55	2.30
±	0.02	0.02	0.02	0.01	0.01	0.01	0.07	0.05	0.08

Supplementary Table 2: NAD(P)H fluorescence decay parameters in wild-type HEK293 cell lines in response to treatments chosen to perturb ATP production.

	Routine	Leak	ETS
wtHEK	20±4	3±2	36±3
NADK+	37±9	6±2	90±10
NADK-	32±9	6±3	70±10

Supplementary Table 3: Oxygen consumption rates (per cell x 10^{-6} pmol s^{-1}) in wild-type and genetically modified HEK293 cell lines under routine (basal), leak (2.5 µM oligomycin) and electron transport system (2 µM FCCP) conditions, adjusted for non-mitochondrial respiration (2.5 µM antimycin).

	Rate
wtHEK	4.5±0.3
NADK+	3.9±0.3
NADK-	3.9±0.1

Supplementary Table 4: Lactate release rates (molecules per cell x 10^7 s^{-1}) of wild-type and genetically modified HEK293 cell lines to indicate glycolytic activity.

	τ_{free} (ns)	α_{bound}	τ_{bound} (ns)
OHC's	0.42	0.26	2.9
±	0.02	0.01	0.1
w/EGCG	0.37	0.21	2.7
±	0.01	0.01	0.1
OPC's	0.40	0.27	3.5
±	0.01	0.01	0.1
w/EGCG	0.35	0.20	2.9
±	0.02	0.01	0.2

Supplementary Table 5: NAD(P)H fluorescence decay parameters in outer hair cells (OHC's) and outer pillar (support) cells (OPC's) of the mammalian cochlea under control conditions and in response to EGCG treatment.

	τ_{free} (ns)	α_{bound}	τ_{bound} (ns)
wtHEK	-0.01±0.02	0.01±0.02	0.1±0.3
NADK+	-0.01±0.02	-0.01±0.01	0.1±0.3
NADK-	0±0.04	0±0.03	0.1±0.4

Supplementary Table 6: Mean differences between parameter values extracted from the same 21x21 pixel region of a cell before and after imaging under the conditions used in this work. The absence of changes in each parameter suggests that the routine imaging conditions used do not introduce artefacts into the results.

	τ_{free} (ns)	α_{bound}	τ_{bound} (ns)
wtHEK	0.05±0.02	0.01±0.01	0.2±0.1
NADK+	0.02±0.01	0.01±0.01	0.2±0.2
NADK-	0.04±0.02	0.03±0.02	0.3±0.2

Supplementary Table 7: Root mean squared differences between parameter values extracted from the same 21x21 pixel region of a cell before and after imaging under the conditions used in this work. These absolute deviations are similar in magnitude to the reported uncertainties of the measurements in supplementary table 1.

Supplementary Notes

Supplementary Note 1. Modelling the signal-to-noise limitations of NAD(P)H FLIM

The Poisson noise inherent in a fluorescence decay measured by the time-correlated single photon counting (TCSPC) method increases proportionally to the square root of the number of photons counted[1]. Increasing the amount of fluorescence collected therefore improves the ratio of signal to noise. Avoiding pulse pile-up effects by limiting the fluorescence detection rate to less than 1% of the repetition rate of the incident laser pulses[2] sets a technical limit on the time scale over which an acceptable signal level can be reached. With 80 MHz excitation in the FLIM setup used in this work, the maximum count rate permitted is 800 kHz. Spread over a 256 x 256 image, this corresponds to an average count rate of approximately 12 counts per pixel per second.

The signal level of a fluorescence decay can be inferred from the number of photon counts in the peak channel, I_0[3]. This can be estimated from the total number of photons counted, I_{total}, by,

$$I_0 = \frac{I_{\text{total}}}{\sum_{i=1}^{n} \sum_{j} \alpha_j \exp\left(-\frac{(i-1)\Delta t}{\tau_j}\right)} \tag{1}$$

where j denotes each species, with amplitude α_j and lifetime τ_j, and Δt is the time spacing between the n bins. With 256 time bins, the double-sum term equals 27 for the lifetime distribution shown in supplementary figure 1a. At the maximum permitted fluorescence count rate, I_0 at each pixel is therefore the total imaging time in seconds multiplied by 0.46.

In a live-cell FLIM experiment, the total imaging time, and therefore the extent to which the contribution of Poisson noise to the measured fluorescence decays can be reduced, is restricted by the requirement to maintain the integrity of the biological sample being imaged[4]. Previously, Tiede and Nichols (2006)[5] observed the generation of highly fluorescent lesions at the onset of cellular damage when imaging NAD(P)H with two-photon microscopy after three minutes of scanning at 20 mW. Acquisition times of between one and three minutes would permit between 30 and 80 counts in the peak bin of the fluorescence decay according to

equation 1. As this is insufficient signal for the accurate fitting of decay curves[1], the fluorescence decay at a given pixel in a FLIM experiment is frequently created from a sum with all surrounding pixels[6,7]. The number of counts in the peak bin of the fluorescence decay located at a pixel of a FLIM image will be increased by an order of magnitude by binning direct neighbours.

In practice, the maximal count rate is unobtainable for live-cell NAD(P)H FLIM due to the low incident laser powers used to maintain sample integrity[4] and the small (0.02) quantum yield of NAD(P)H. As a result, the average fluorescence count rate while imaging was approximately 40 kHz in this work, 20 times smaller than the maximal rate. Binning of direct neighbours resulted in only 50 to 75 counts in the peak bin of fluorescence decays within the darkest regions of the cell (the nuclei). Binning was therefore increased to 24 surrounding pixels (five by five square) across the whole image. This resulted in a more suitable 150 to 200 counts in the peak bin of dark regions and 500 to 600 in the brightest regions. These values were also used to define the range of I_0 values (100-1000 peak counts) used in computational simulations aimed at investigating how the simplified fluorescence decays measured using FLIM reflect an underlying distribution of NAD(P)H lifetimes, as their validity rests on the simulated fluorescence decays possessing a similar signal to noise ratio as those measured using NAD(P)H FLIM.

To model the NAD(P)H fluorescence decays measured using live-cell FLIM, multiexponential decay functions were simulated in MATLAB. Based on the results of Visser et al. (1981)[8], 0.3 ns and 0.7 ns components were present at a 7:3 ratio to represent the biexponential fluorescence decay of free NAD(P)H in solution. From the work of Skala et al. (2007)[9], the proportion of enzyme-bound species present in the decay, α_{bound}, was initially set at 0.3. Six components with fluorescence lifetimes evenly spaced between 1 ns and 6 ns represented the range of bound NAD(P)H lifetimes reported in the literature. Each bound species was initially present in equal proportion ($\alpha_{\text{bound}}/6 = 0.05$). Datasets were binned at 0.0488 ns intervals and convolved with a Gaussian instrument response function with 0.1 ns full width at half maximum to represent the operating parameters of the lifetime imaging system used in this work. The convolved decay was scaled such that a predefined number of counts were present in the peak bin and a baseline of 10 counts was added as uncorrelated background noise.

Poisson noise was added to each bin using the poissrnd() MATLAB function. The generated data were subsequently fit to fluorescence decay models using the FLUOFIT iterative reconvolution package[10].

With 1000 counts in the peak channel a single exponential decay model was a poor fit for the data (supplementary figure 1b). However, increasing the number of lifetime components to two (supplementary figure 1c) improved the quality-of-fit parameter χ_r^2 from 7.1 to 1.0, suggesting a perfect fit. By generating 100 unique decays at signal levels between 100 and 1000 peak counts and repeating this fitting process, it was observed that a monoexponential model became a poorer description of the data as the signal level was increased. However, across the whole range of signal levels possible with NAD(P)H FLIM of live tissue, a biexponential model was a good fit (supplementary figure 1d). Addition of more lifetime components could not improve the quality of the biexponential fits further within this range, explaining the presence of only two lifetime components in the pixel-wise NAD(P)H fluorescence decays measured using FLIM.

The mean values of τ_{free} and τ_{bound} extracted from the simulated NAD(P)H fluorescence decays remained close to the amplitude-weighted mean values of the free and enzyme-bound lifetime components of the synthetic data; 0.42 ns and 3.5 ns respectively (supplementary figure 1e) although, at the lowest signal levels, the standard deviation of the 100 lifetime values extracted increased dramatically (supplementary figure 1i). This resulted from shallowing of the support plane of each parameter with decreased signal (supplementary figures 1f-h), observed by repeating the fitting process while holding τ_{free}, α_{bound} or τ_{bound} constant at a range of values close to the region of minimum χ_r^2. The support planes of the simulated NAD(P)H fluorescence decays were similar to those of datasets simulated with only two species with lifetimes of 0.42 ns and 3.5 ns. This confirmed that the wide support plane resulted from the decreased signal-to-noise rather than the introduction of a distribution of decay components. Indeed, application of this method to an NAD(P)H fluorescence decay measured in wild-type HEK293 cells produced a similar result. While the two lifetimes best fit to the simulated NAD(P)H fluorescence decays were in agreement with the amplitude-weighted mean values of the free and enzyme-bound species present, confirming the validity of labelling these components τ_{free} and τ_{bound}, the values of α_{bound} consistently underestimated the true fraction of bound species input into the lifetime distribution. This

could have been due to a portion of the enzyme-bound fluorescence being assigned to the free amplitude, causing the parameter correlation shown in supplementary figure 1j.

As repeated fluorescence decay measurements on the same population will sample the local minimum of the associated χ_r^2 surface, a larger spread of parameter values would be expected to be obtained with 1,000 peak channel counts compared to 10,000. This constitutes a problem for TCSPC studies in which conclusions are drawn from comparisons between single fluorescence decay measurements. However, in this work, the 10^3-10^4 unique sets of parameters extracted from the fluorescence decays located within a region of interest of the FLIM image were averaged to report an overall value for that region. The simulations performed here show that averaging across a region of interest of a FLIM image provides a reliable measure of the mean fluorescence lifetimes of the free and enzyme-bound NAD(P)H species present there, with the spread of these measurements reflecting the width of the support plane and thus the precision of the support plane minimum.

Given the hypothesis that enzyme-bound NADPH is associated with a larger fluorescence lifetime inside the cell than enzyme-bound NADH, further computational simulations were performed in order to understand how τ_{bound} would respond to changes in the relative concentrations of the two cofactors. The behaviour of this parameter following changes in the proportion of two enzyme-bound NAD(P)H components with differing lifetimes was investigated. Simulated fluorescence decays were generated in which the amplitudes of hypothetical 2 ns and 5 ns components, α_{NADH} and α_{NADPH} respectively, were varied with respect to one-another at a series of fixed α_{bound} values (supplementary figure 1k). The measured τ_{bound} decreased linearly as α_{NADH} was increased from zero to α_{bound}, as shown in supplementary figure 1l. The slight variation in τ_{bound} values between each dataset at $\alpha_{\text{NADH}} = 0$ and $\alpha_{\text{NADH}} = \alpha_{\text{bound}}$ implies a small correlation between this parameter and α_{bound}. At $\alpha_{\text{NADH}} = 0$, the magnitude of this variation was $\Delta\tau_{\text{bound}}/\Delta\alpha_{\text{bound}} \approx 1.5$, decreasing to ≈ 0.5 at $\alpha_{\text{NADH}} = \alpha_{\text{bound}}$. Based on this model, the maximum possible contribution of parameter correlation to changes in τ_{bound} is therefore 0.08 ns for EGCG treatment of NADK+ cells, 0.06 ns for the difference in α_{bound} between NADK+ and NADK- cells and 0.08 ns for the difference in α_{bound} between the cytosol and nucleus of

wild type HEK293 cells. Comparison of these values to the measured τ_{bound} differences of 0.5 ns, 1.1 ns and 0.4 ns respectively and their small size relative to the uncertainty already on these measurements demonstrates that correlation between τ_{bound} and α_{bound} is sufficiently small to have a negligible effect on the conclusions of this work.

Supplementary Note 2. Estimation of NADH- and NADPH-associated fluorescence lifetimes

By making the assumption that NADH and NADPH possess distinct fluorescence lifetimes when bound to enzymes inside the cell, τ_{NADH} and τ_{NADPH} respectively, the computational simulations of the previous section and the concentrations of NADH and NADPH in the NADK+ and NADK- cells previously reported by Pollack et al. (2007[11]) were used to form a mathematical model to aid the explanation of the behaviour of τ_{bound}. By assigning the minimum and maximum lifetimes measured for each α_{bound} value as τ_{NADH} and τ_{NADPH}, the equation for the straight lines in supplementary figure 11 could be written,

$$\tau_{\mathrm{bound}} = \left(\frac{\tau_{\mathrm{NADH}} - \tau_{\mathrm{NADPH}}}{\alpha_{\mathrm{bound}}}\right)\alpha_{\mathrm{NADH}} + \tau_{\mathrm{NADPH}} \qquad (2)$$

This equation could be rewritten in terms of the concentrations of bound NADPH and NADH contributing to the fluorescence decay as,

$$\frac{\tau_{\mathrm{bound}} - \tau_{\mathrm{NADH}}}{\tau_{\mathrm{NADPH}} - \tau_{\mathrm{NADH}}} = \frac{[\mathrm{NADPH}]_{\mathrm{bound}}}{[\mathrm{NADH}]_{\mathrm{bound}} + [\mathrm{NADPH}]_{\mathrm{bound}}} \qquad (3)$$

As no *a priori* knowledge of the proportion of bound NAD(P)H consisting of NADH or NADPH exists, the approximation had to be made that the fractions of NADH and NADPH present in the enzyme bound population were similar to the overall populations of each cofactor present. Equation 3 was then written,

$$\tau_{\mathrm{bound}} = \left(\tau_{\mathrm{NADPH}} - \tau_{\mathrm{NADH}}\right)\frac{[\mathrm{NADPH}]}{[\mathrm{NADH}] + [\mathrm{NADPH}]} + \tau_{\mathrm{NADH}} \qquad (4)$$

Writing the ratios of NADPH to NADH in the NADK+ and NADK- cells as R^{+} and R^{-} respectively, with associated enzyme-bound fluorescence lifetimes in these cells measured as $\tau^{+}_{\mathrm{bound}}$ and $\tau^{-}_{\mathrm{bound}}$, the NADH- and NADPH-associated enzyme-bound fluorescence lifetimes were given by,

$$\tau_{\mathrm{NADH}} = \frac{\tau^{-}_{\mathrm{bound}} R^{+}\left(1 + R^{-}\right) - \tau^{+}_{\mathrm{bound}} R^{-}\left(1 + R^{+}\right)}{R^{+} - R^{-}} \qquad (5)$$

$$\tau_{\mathrm{NADPH}} = \frac{\tau_{\mathrm{bound}}^{+}\left(1+R^{+}\right)-\tau_{\mathrm{bound}}^{-}\left(1+R^{-}\right)}{R^{+}-R^{-}} \tag{6}$$

The measured values of τ_{bound} differed between the mitochondrial, cytosolic and nuclear compartments. However, cofactor concentrations were available only for the cell as a whole, so this had to be taken into account by calculating effective whole-cell enzyme-bound fluorescence lifetimes, $\tau_{\mathrm{bound}}^{\mathrm{eff},+}$ and $\tau_{\mathrm{bound}}^{\mathrm{eff},-}$, to replace $\tau_{\mathrm{bound}}^{+}$ and $\tau_{\mathrm{bound}}^{-}$ in equations 5 and 6. These were calculated as,

$$\tau_{\mathrm{bound}}^{\mathrm{eff}} = \frac{A_{\mathrm{mito}}C_{\mathrm{mito}}\tau_{\mathrm{bound,mito}} + A_{\mathrm{cyto}}C_{\mathrm{cyto}}\tau_{\mathrm{bound,cyto}} + A_{\mathrm{nuc}}C_{\mathrm{nuc}}\tau_{\mathrm{bound,nuc}}}{A_{\mathrm{mito}}C_{\mathrm{mito}} + A_{\mathrm{cyto}}C_{\mathrm{cyto}} + A_{\mathrm{nuc}}C_{\mathrm{nuc}}} \tag{7}$$

where A is the area of the mitochondrial, cytosolic or nuclear compartments in each image with total NAD(P)H concentration C, given by[12],

$$C = k\frac{\langle I_{\mathrm{total}}\rangle}{(1-\alpha_{\mathrm{bound}})\tau_{\mathrm{free}} + \alpha_{\mathrm{bound}}\tau_{\mathrm{bound}}} \tag{8}$$

where $\langle I_{\mathrm{total}}\rangle$ is the fluorescence intensity in the subcellular region and k is an arbitrary constant. The effective enzyme-bound fluorescence lifetimes relate to the whole-cell R values by,

$$R = \frac{[\mathrm{NADPH}]}{[\mathrm{NADH}]} = \frac{\tau_{\mathrm{bound}}^{\mathrm{eff}} - \tau_{\mathrm{NADH}}}{\tau_{\mathrm{NADPH}} - \tau_{\mathrm{bound}}^{\mathrm{eff}}} \tag{9}$$

It can be shown that substitution of equation 7 into equation 9 gives the correct expression for the total, uncompartmentalised NADPH to NADH ratio.

HPLC of the pyridine nucleotide contents of the NADK+ and NADK- cells previously reported by Pollack et al. (2007)[11] gave values of R^{+} and R^{-} as 2.3 and 0.6 respectively, leading to values of τ_{NADH} and τ_{NADPH}, using equations 5 to 8, of 1.5±0.2 ns and 4.4±0.2 ns respectively. The fluorescence intensity $\langle I_{\mathrm{total}}\rangle$ (total photons counted) can be introduced to separate this ratiometric quantity into absolute changes[12,13], giving,

$$[\text{NADH}] = k\left(\frac{4.4 - \tau_{\text{bound}}(\text{ns})}{2.9}\right)\frac{\langle I_{\text{total}}\rangle}{(1-\alpha_{\text{bound}})\tau_{\text{free}} + \alpha_{\text{bound}}\tau_{\text{bound}}} \quad (10)$$

$$[\text{NADPH}] = k\left(\frac{\tau_{\text{bound}}(\text{ns}) - 1.5}{2.9}\right)\frac{\langle I_{\text{total}}\rangle}{(1-\alpha_{\text{bound}})\tau_{\text{free}} + \alpha_{\text{bound}}\tau_{\text{bound}}} \quad (11)$$

While the sensitivity of α_{bound} to EGCG treatment in NADK+ but not NADK- could be expected given the relative abundance of the two cofactors in the enzyme bound populations of the two cell lines, τ_{bound} also remained constant in the NADK- cells upon EGCG application. Such a result suggested that the magnitude of the response of τ_{bound} to EGCG treatment was proportional to the initial ratio of NADPH to NADH present in the cells. The model developed here accounts for this phenomenon due to the non-linear dependence on the ratio of NADPH to NADH causing τ_{bound} to be less sensitive to changes in this ratio when it is initially top-heavy or bottom-heavy (supplementary figure 1n-o).

Supplementary Note 3. Validation of quantitative model using EGCG treatment data

The above model allows estimates of the intracellular concentrations of bound NADH and bound NADPH to be quantified in arbitrary units. To demonstrate this procedure, the effect of EGCG treatment on the bound NADH and NADPH concentrations in NADK+ cells was calculated. Intensities were extracted from the average total photon counts in each subcellular location in each image acquired. These values were then combined with the corresponding NAD(P)H FLIM parameters of each dataset using equations 10 and 11. This provided 9 bound NADH concentrations and 9 bound NADPH concentrations in each subcellular compartment both before and after EGCG treatment. Taking means of these values with k=1,710 (chosen to fix the cytosolic NADH concentration at 1 au), the concentration of bound NADH remained constant upon EGCG application at 1.4±0.4 au, 1.0±0.4 au and 1.8±0.2 au in the mitochondria, cytosol and nuclei respectively, while the concentration of bound NADPH decreased from 4±2 au to 1.6±0.5 au in the mitochondria, 4±1 au to 1.3±0.4 au in the cytosol and 2±1 au to 0.7±0.2 au in the nuclei ($P=0.004$, Wilcoxon signed-rank test, $n=9$). That this simple model could replicate the known function of EGCG from the measured NAD(P)H fluorescence intensity and decay parameters lends support to its validity.

Supplementary Note 4. Redox state of nuclear and cytosolic NAD+/NADH and NADP+/NADPH pools

It can be assumed that diffusion of small molecules through the nuclear pores equilibriates the total concentrations of the NAD+/NADH and NADP+/NADPH pools in the cytosol and nucleus[14]. The differing proportions of NADH and NADPH in the two compartments must then result from differing redox states of each pool. Expressing this statement mathematically,

$$[\mathrm{NAD+}]_{\mathrm{cyto}} + [\mathrm{NADH}]_{\mathrm{cyto}} = [\mathrm{NAD+}]_{\mathrm{nuc}} + [\mathrm{NADH}]_{\mathrm{nuc}} \tag{12}$$

$$[\mathrm{NADP+}]_{\mathrm{cyto}} + [\mathrm{NADPH}]_{\mathrm{cyto}} = [\mathrm{NADP+}]_{\mathrm{nuc}} + [\mathrm{NADPH}]_{\mathrm{nuc}} \tag{13}$$

Substitution of the redox ratios, written as,

$$D_{\mathrm{cyto}} = \frac{[\mathrm{NAD+}]_{\mathrm{cyto}}}{[\mathrm{NADH}]_{\mathrm{cyto}}} \tag{14}$$

$$D_{\mathrm{nuc}} = \frac{[\mathrm{NAD+}]_{\mathrm{nuc}}}{[\mathrm{NADH}]_{\mathrm{nuc}}} \tag{15}$$

$$P_{\mathrm{cyto}} = \frac{[\mathrm{NADP+}]_{\mathrm{cyto}}}{[\mathrm{NADPH}]_{\mathrm{cyto}}} \tag{16}$$

$$P_{\mathrm{nuc}} = \frac{[\mathrm{NADP+}]_{\mathrm{nuc}}}{[\mathrm{NADPH}]_{\mathrm{nuc}}} \tag{17}$$

leads to,

$$[\mathrm{NADH}]_{\mathrm{cyto}}(1 + D_{\mathrm{cyto}}) = [\mathrm{NADH}]_{\mathrm{nuc}}(1 + D_{\mathrm{nuc}}) \tag{18}$$

$$[\mathrm{NADPH}]_{\mathrm{cyto}}(1 + P_{\mathrm{cyto}}) = [\mathrm{NADPH}]_{\mathrm{nuc}}(1 + P_{\mathrm{nuc}}) \tag{19}$$

Rearranging these equations and using equations 10 and 11 with the experimentally obtained fluorescence intensity and decay parameters to calculate the concentrations of NADH and NADPH in the cytosol and nucleus of wild-type HEK293 cells gives,

$$\frac{[\text{NADH}]_{\text{nuc}}}{[\text{NADH}]_{\text{cyto}}} = \frac{(1 + D_{\text{cyto}})}{(1 + D_{\text{nuc}})} = 1 \tag{20}$$

$$\frac{[\text{NADPH}]_{\text{nuc}}}{[\text{NADPH}]_{\text{cyto}}} = \frac{(1 + P_{\text{cyto}})}{(1 + P_{\text{nuc}})} = 0.57 \tag{21}$$

Equation 20 implies that the redox ratios of the cytosolic and nuclear NAD+/NADH pools are equal. However, rearranging equation 21 gives,

$$P_{\text{nuc}} = 1.75 P_{\text{cyto}} + 0.75 \tag{22}$$

So, $P_{\text{nuc}} > P_{\text{cyto}}$, implying that the NADP+/NADPH pool is maintained in a more oxidised state in the nucleus than in the cytosol. A BRENDA enzyme localisation search indicated that glyceraldehyde 3-phosphate dehydrogenase (EC 1.2.1.12), the NADH-producing enzyme of the glycolytic pathway, can be found in the nucleus. However, the NADPH-producing enzymes of the pentose phosphate pathway, glucose 6-phosphate dehydrogenase (EC 1.1.1.49) and 6-phosphogluconate dehydrogenase (EC 1.1.1.44), are only found in the cytosol. This shortfall in NADPH production in the nucleus supports the predictions of our model.

Supplementary Note 5. Spectral controls

A range of wavelengths have previously been used for two-photon excitation of NAD(P)H in live cells and tissues, ranging from 710 nm[15] to 780 nm[9]. The two-photon absorption spectrum of NADH peaks at 700 nm with an absolute cross section of approximately 4 GM[16]. As such, excitation at this wavelength is desirable in order to decrease incident powers or imaging times while maintaining signal. One caveat to the use of this wavelength lies in the amount of other sources of autofluorescence excited at 700 nm relative to other wavelengths. Previously, Zipfel et al. (2003)[16] investigated the two-photon absorption and emission properties of a number of endogenous fluorophores, including NADH, folic acid, vitamins A and D, pyridoxine and flavin. Notably, the two-photon action cross section of flavin was shown to be at least 10-times greater than any other autofluorescent compound across a wide spectral range, highlighting the importance of filtering out fluorescence at 530±30nm for NAD(P)H imaging. Based on the reported data, if the array of autofluorescent compounds characterised were present in a solution at equal concentration, the total signal detected through the 460±25nm emission filter used in the present work would consist of 54% NAD(P)H with 700 nm excitation, compared to 51% and 47% at 720 nm and 740 nm respectively. However, many of the species contaminating the signal will be present at significantly lower concentrations than NAD(P)H[17]. Repeating these calculations upon approximating values for the biological concentrations of these metabolites from the Human Metabolome Database[18] results in 95% of the total fluorescence collected resulting from NAD(P)H at 700 nm, 720 nm and 740 nm.

While the above discussion gives confidence in the specificity of the excitation and emission conditions used in this work, concentrations of endogenous fluorophores may be tissue-specific and it is impossible to rule out potential contributions from other sources. As such, the levels of contaminating autofluorescence observed in HEK293 cells with 460±25 nm emission filtering and 700 nm excitation were compared to those with the alternative wavelengths of 720 nm and 740 nm. Application of 1 μM FCCP to the cells caused the NAD(P)H-specific fraction of the total fluorescence to decrease to a fraction γ of its initial value. Importantly, while the total amount of NAD(P)H fluorescence and additional autofluorescence would vary across the different excitation wavelengths, γ would remain constant, allowing the levels of autofluorescence to be compared. This

experimental scheme is shown in supplementary figure 4a. The fraction of the total fluorescence at excitation wavelength λ consisting of autofluorescence A_λ can be written,

$$\frac{A_\lambda}{I_\lambda^{\text{before}}} = \frac{I_\lambda^{\text{after}} - \gamma I_\lambda^{\text{before}}}{I_\lambda^{\text{before}}(1 - \gamma)} \tag{23}$$

where $I_\lambda^{\text{before}}$ and I_λ^{after} are the total fluorescence signals measured prior-to and following the application of FCCP. Nine readings of $I_\lambda^{\text{before}}$ and I_λ^{after} were taken for each excitation wavelength and the results are plotted in supplementary figure 4b using equation 23 for all possible values of γ. It can be seen that no additional contaminating autofluorescence is evident with 700 nm excitation compared to 720 nm or 740 nm.

Supplementary Note 6. Photodamage controls

Photobleaching has previously been linked to the onset of photodamage with NAD(P)H imaging[5]. As such, fluorescence count rates were monitored for constancy during image acquisition. The microscopy parameters applied in this work (pixel dwell period, laser power, imaging time) were chosen to allow the acquisition of sufficient signal in the absence of photobleaching. Control experiments were also performed to ensure that no laser-induced metabolic or photophysical phenomena were taking place inside the cells over the imaging period, causing the NAD(P)H fluorescence decay parameters to vary over the acquisition time. To do this, FLIM images of a group of HEK293 cells were acquired for 10 seconds both before and after imaging under routine conditions. Spatial resolution was sacrificed, with a 21x21 pixel region of interest in the cell cluster defined, within which the counts from each image were aggregated to produce two fluorescence decays with similar signal-to-noise levels as those acquired in the main study. Upon fitting, values for τ_{free}, α_{bound} and τ_{bound} were obtained for the same region of interest both before and after routine acquisition, allowing any variation in their values to be assessed. This procedure was performed 9 times, with results displayed in supplementary tables 6 and 7. It can be seen that the mean differences between the parameters at the start and end of routine imaging were close to zero. The root mean squared of these differences were similar to the standard deviations of the measurements themselves. This indicated that any variation could not be distinguished from the expected spread of values obtained. Together, these results demonstrate that changes in FLIM parameters during imaging period would not affect the measurements made within this study and the conclusions drawn from them.

Supplementary References

1. Köllner, M. & Wolfrum, J. How many photons are necessary for fluorescence-lifetime measurements? *Chem. Phys. Lett.* **200,** 199–204 (1992).

2. Salthammer, T. Numerical simulation of pile-up distorted time-correlated single photon counting (TCSPC) data. *J. Fluoresc.* **2,** 23–27 (1992).

3. James, D. R. & Ware, W. R. A fallacy in the interpretation of fluorescence decay parameters. *Chem. Phys. Lett.* **120,** 455–459 (1985).

4. Hopt, A. & Neher, E. Highly Nonlinear Photodamage in Two-Photon Fluorescence Microscopy. *Biophys. J.* **80,** 2029–2036 (2001).

5. Tiede, L. M. & Nichols, M. G. Photobleaching of reduced nicotinamide adenine dinucleotide and the development of highly fluorescent lesions in rat basophilic leukemia cells during multiphoton microscopy. *Photochem Photobiol* **82,** 656–664 (2006).

6. Bird, D. K. *et al.* Metabolic Mapping of MCF10A Human Breast Cells via Multiphoton Fluorescence Lifetime Imaging of the Coenzyme NADH. *Cancer Res.* **65,** 8766–8773 (2005).

7. Becker, W. *et al.* Fluorescence lifetime imaging by time-correlated single-photon counting. *Microsc. Res. Tech.* **63,** 58–66 (2004).

8. Visser, A. J. W. G. & Hoek, A. van. The fluorescence decay of reduced nicotinamides in aqueous solution after excitation with a UV-mode locked locked Ar ion laser. *Photochem Photobiol* **33,** 35–40 (1981).

9. Skala, M. C. *et al.* In vivo multiphoton fluorescence lifetime imaging of protein-bound and free nicotinamide adenine dinucleotide in normal and precancerous epithelia. *J. Biomed. Opt.* **12,** 24014 (2007).

10. Enderlein, J. & Erdmann, R. Fast fitting of multi-exponential decay curves. *Opt. Commun.* **134,** 371–378 (1997).

11. Pollak, N., Niere, M. & Ziegler, M. NAD kinase levels control the NADPH concentration in human cells. *J. Biol. Chem.* **282,** 33562–33571 (2007).

12. Xu, C. & Webb, W. W. Measurement of two-photon excitation cross sections of molecular fluorophores with data from 690 to 1050 nm. *J. Opt. Soc. Am. B* **13,** 481–491 (1996).

13. Yu, Q. & Heikal, A. A. Two-photon autofluorescence dynamics imaging reveals sensitivity of intracellular NADH concentration and conformation to cell physiology at the single-cell level. *J. Photochem. Photobiol. B* **95,** 46–57 (2009).

14. Zhang, Q., Piston, D. W. & Goodman, R. H. Regulation of corepressor function by nuclear NADH. *Science (80).* **295,** 1895–1897 (2002).

15. Kasischke, K. A. *et al.* Two-photon NADH imaging exposes boundaries of oxygen diffusion in cortical vascular supply regions. *J. Cereb. Blood Flow Metab.* **31,** 68–81 (2011).

16. Zipfel, W. R. *et al.* Live tissue intrinsic emission microscopy using multiphoton-excited native fluorescence and second harmonic generation. *Proc. Natl. Acad. Sci. U. S. A.* **100,** 7075–80 (2003).

17. Aubin, J. E. Autofluorescence of viable cultured mammalian cells. *J Histochem Cytochem* **27,** 36–43 (1979).

18. Wishart, D. S. *et al.* HMDB 3.0--The Human Metabolome Database in 2013. *Nucleic Acids Res.* **41,** D801–7 (2013).
